# Supplementary material for: Causal relationship between Helicobacter pylori antibodies and gastroesophageal reflux disease (GERD): A mendelian study
Source: PLoS One. 2023 Dec 11;18(12):e0294771. doi: 10.1371/journal.pone.0294771 (PMC10712878; doi:10.1371/journal.pone.0294771)
Supplement: S1 Table — Instrumental SNPs of GERD. (DOCX) [file pone.0294771.s001.docx]

## Supplementary Table

| **SNP** | **Chr: BP** | **Effect allele** | **Other allele** | **MAF** | **P-value** |
| --- | --- | --- | --- | --- | --- |
| rs116421363 | 1:181820561 | C | A | 0.363202 | 2.68E-06 |
| rs75170215 | 3:25908239 | C | T | 0.02057 | 2.02E-06 |
| rs75740599 | 4:159123611 | A | G | 0.252886 | 4.27E-06 |
| rs571061419 | 6:162760485 | A | G | 0.074197 | 4.21E-06 |
| rs6530847 | 8:15111565 | A | T | 0.391211 | 3.41E-06 |
| rs4268452 | 10:81319354 | T | C | 0.071203 | 4.62E-06 |
| rs117827497 | 11:105212623 | G | A | 0.030462 | 3.10E-06 |
| rs138363822 | 11:1606147 | G | A | 0.426282 | 4.30E-06 |
| rs117537486 | 11:94351805 | C | G | 0.03072 | 4.22E-06 |
| rs149747348 | 12:98890443 | C | G | 0.013186 | 3.92E-06 |
| rs56264437 | 13:102123887 | A | G | 0.603666 | 1.99E-06 |
| rs11858369 | 15:90495509 | G | A | 0.068381 | 4.18E-07 |
| rs553266653 | 16:46420007 | C | A | 0.033679 | 4.04E-06 |
| rs118006294 | 16:10974221 | C | T | 0.050367 | 3.04E-06 |

**S1 Table. Mendelian randomization analysis of Hp and GERD.** Instrumental SNPs of GERD.
