# Supplementary material for: Causal relationship between Helicobacter pylori antibodies and gastroesophageal reflux disease (GERD): A mendelian study
Source: PLoS One. 2023 Dec 11;18(12):e0294771. doi: 10.1371/journal.pone.0294771 (PMC10712878; doi:10.1371/journal.pone.0294771)
Supplement: S1 Fig — (A) Hp-CagA and GERD; (B) Hp-Catalase and GERD; (C) Hp-GroEL and GERD; (D) Hp-IgG and GERD; (E) Hp-OMP and GERD; (F) Hp-UREA and GERD; (G) Hp-VacA and GERD. (DOCX) [file pone.0294771.s002.docx]

## Supplementary Figures

**
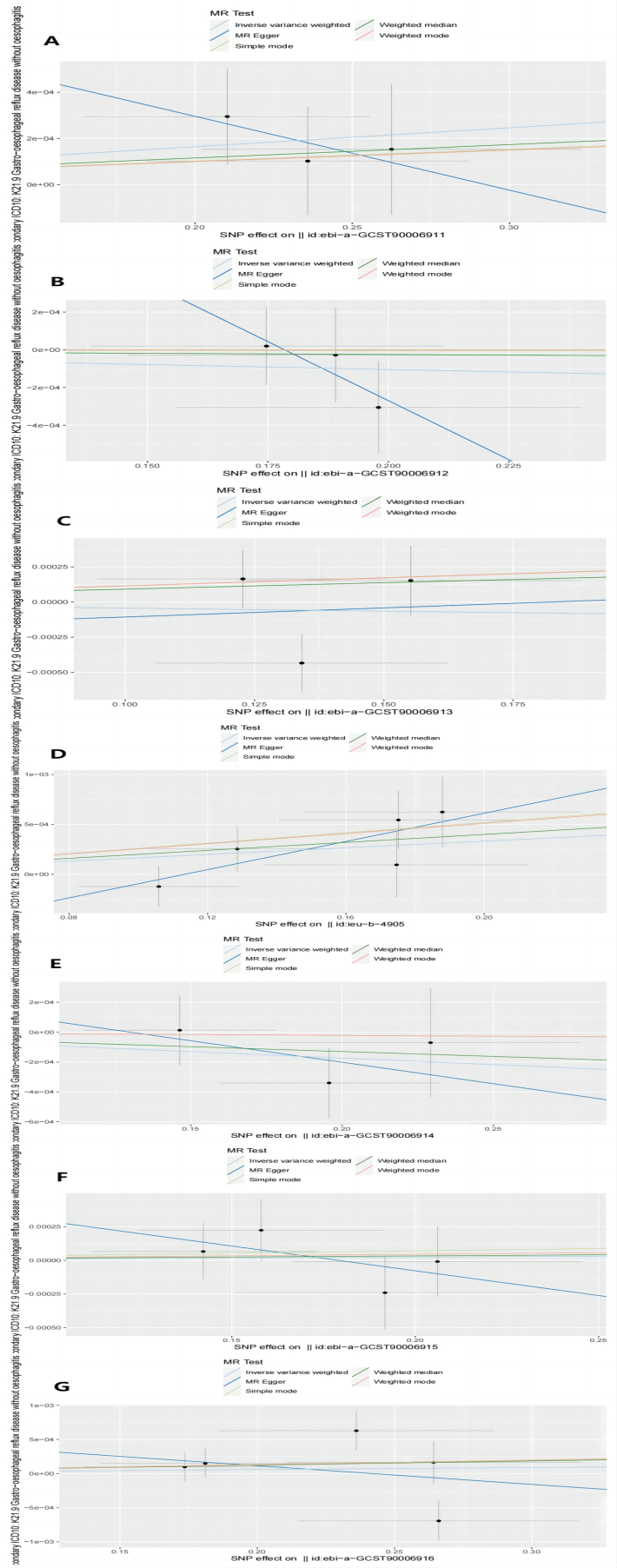
**

**S1 Fig. Scatter plot.** (A) Hp-CagA and GERD; (B) Hp-Catalase and GERD; (C) Hp-GroEL and GERD; (D) Hp-IgG and GERD; (E) Hp-OMP and GERD; (F) Hp-UREA and GERD; (G) Hp-VacA and GERD**.**
