# Supplementary material for: Causal relationship between Helicobacter pylori antibodies and gastroesophageal reflux disease (GERD): A mendelian study
Source: PLoS One. 2023 Dec 11;18(12):e0294771. doi: 10.1371/journal.pone.0294771 (PMC10712878; doi:10.1371/journal.pone.0294771)
Supplement: Attachment — Submitted filename: PONE-D-23-28111_reviewer-REVISION.pdf [file pone.0294771.s006.pdf]

PLOS ONE

Causal Relationship Between Helicobacter pylori Antibodies and gastroesophageal reflux disease (GERD): A Mendelian Study

--Manuscript Draft--

**Manuscript Number:**	PONE-D-23-28111
**Article Type:**	Research Article
**Full Title:**	Causal Relationship Between Helicobacter pylori Antibodies and gastroesophageal reflux disease (GERD): A Mendelian Study
**Short Title:**	Causal Relationship Between Helicobacter pylori Antibodies and gastroesophageal reflux disease (GERD)
**Corresponding Author:**	Zhihong Li<br>Beijing University of Chinese Medicine Affiliated Dongzhimen Hospital<br>Beijing, CHINA
**Keywords:**	Mendelian randomization; Helicobacter pylori; gastroesophageal reflux disease; bidirectional; causal
**Abstract:**	Background<br><br>Observational studies have indicated that both Helicobacter pylori infection and the presence of Helicobacter pylori antibodies may increase the risk of gastroesophageal reflux disease (GERD). However, the exact association between Helicobacter pylori antibodies and the occurrence of GERD remains largely unresolved. Therefore, this two-sample Mendelian randomization (MR) study aims to investigate the causal relationship between Helicobacter pylori infection and GERD.<br><br>Methods<br><br>This study encompassed seven different specific protein antibodies targeting Helicobacter pylori and utilized a genome-wide association study (GWAS) on GERD. MR analysis was conducted to assess the causal relationship between Helicobacter pylori antibodies and the development of GERD.<br><br>Results<br><br>Genetically predicted serum levels of Helicobacter pylori IgG antibodies were positively associated with an increased risk of GERD (odds ratio [OR] = 1.001, 95% CI 1.000-1.003, P = 0.043). No causal relationship was found between other Helicobacter pylori antibodies and gastroesophageal reflux disease.<br><br>Conclusion<br><br>The outcomes derived from our two-sample Mendelian randomization analysis demonstrate a discernible link between the levels of Helicobacter pylori IgG antibodies and an augmented susceptibility to GERD. However, it is imperative to expand the sample size further in order to corroborate the correlation between Helicobacter pylori infection and GERD.
**Order of Authors:**	Jiaqin Chen
	Junning Zhang
	Xiaolu Ma
	Yuehan Ren
	Yi Tang
	Zhongmian Zhang
	Wangyu Ye
	Xiyan Zhang

Powered by Editorial Manager® and ProduXion Manager® from Aries Systems Corporation

	Zili Lin
	Lan Wang
	Zhihong Li
**Additional Information:**	
**Question**	**Response**
**Financial Disclosure**<br><br>Enter a financial disclosure statement that describes the sources of funding for the work included in this submission. Review the submission guidelines for detailed requirements. View published research articles from *PLOS ONE* for specific examples.<br><br>This statement is required for submission and **will appear in the published article** if the submission is accepted. Please make sure it is accurate.<br><br>**Unfunded studies**<br>Enter: *The author(s) received no specific funding for this work.*<br><br>**Funded studies**<br>Enter a statement with the following details:<br>• Initials of the authors who received each award<br>• Grant numbers awarded to each author<br>• The full name of each funder<br>• URL of each funder website<br>• Did the sponsors or funders play any role in the study design, data collection and analysis, decision to publish, or preparation of the manuscript?<br>• **NO** - Include this sentence at the end of your statement: *The funders had no role in study design, data collection and analysis, decision to publish, or preparation of the manuscript.*<br>• **YES** - Specify the role(s) played.<br><br>* typeset	Funding<br>Transverse project of Dongzhimen Hospital of Beijing University of Traditional Chinese Medicine: Improvement effect of Lactobacillus plantarum HCS-001 on gastric discomfort symptoms in Helicobacter pylori infected patients (No. HX-DZM-202269).
**Competing Interests**<br><br>Use the instructions below to enter a competing interest statement for this submission. On behalf of all authors, disclose any competing interests that could be perceived to bias this	Competing interests<br>None declared.

work—acknowledging all financial support and any other relevant financial or non-financial competing interests.<br><br>This statement is required for submission and **will appear in the published article** if the submission is accepted. Please make sure it is accurate and that any funding sources listed in your Funding Information later in the submission form are also declared in your Financial Disclosure statement.<br><br>View published research articles from *PLOS ONE* for specific examples.<br><br>**NO authors have competing interests**<br><br>Enter: *The authors have declared that no competing interests exist.*<br><br>**Authors with competing interests**<br><br>Enter competing interest details beginning with this statement:<br><br>*I have read the journal's policy and the authors of this manuscript have the following competing interests: [insert competing interests here]*<br><br>* typeset	
**Ethics Statement**<br><br>Enter an ethics statement for this submission. This statement is required if the study involved:<br><br>• Human participants<br>• Human specimens or tissue<br>• Vertebrate animals or cephalopods<br>• Vertebrate embryos or tissues<br>• Field research<br><br>Write "N/A" if the submission does not require an ethics statement.	Ethics approval and consent to participate<br>Not required.

General guidance is provided below. Consult the submission guidelines for detailed instructions. **Make sure that all information entered here is included in the Methods section of the manuscript.**<br><br>**Format for specific study types**<br><br>**Human Subject Research (involving human participants and/or tissue)**<br>• Give the name of the institutional review board or ethics committee that approved the study<br>• Include the approval number and/or a statement indicating approval of this research<br>• Indicate the form of consent obtained (written/oral) or the reason that consent was not obtained (e.g. the data were analyzed anonymously)<br><br>**Animal Research (involving vertebrate animals, embryos or tissues)**<br>• Provide the name of the Institutional Animal Care and Use Committee (IACUC) or other relevant ethics board that reviewed the study protocol, and indicate whether they approved this research or granted a formal waiver of ethical approval<br>• Include an approval number if one was obtained<br>• If the study involved *non-human primates*, add *additional details* about animal welfare and steps taken to ameliorate suffering<br>• If anesthesia, euthanasia, or any kind of animal sacrifice is part of the study, include briefly which substances and/or methods were applied<br><br>**Field Research**<br><br>Include the following details if this study involves the collection of plant, animal, or other materials from a natural setting:<br>• Field permit number<br>• Name of the institution or relevant body that granted permission	
**Data Availability**<br><br>Authors are required to make all data underlying the findings described fully	Yes - all data are fully available without restriction

Powered by Editorial Manager® and ProduXion Manager® from Aries Systems Corporation

available, without restriction, and from the time of publication. PLOS allows rare exceptions to address legal and ethical concerns. See the PLOS Data Policy and FAQ for detailed information.<br><br>A Data Availability Statement describing where the data can be found is required at submission. Your answers to this question constitute the Data Availability Statement and **will be published in the article**, if accepted.<br><br>**Important:** Stating 'data available on request from the author' is not sufficient. If your data are only available upon request, select 'No' for the first question and explain your exceptional situation in the text box.<br><br>Do the authors confirm that all data underlying the findings described in their manuscript are fully available without restriction?	
**Describe where the data may be found in full sentences. If you are copying our sample text, replace any instances of XXX with the appropriate details.**<br><br>• If the data are **held or will be held in a public repository**, include URLs, accession numbers or DOIs. If this information will only be available after acceptance, indicate this by ticking the box below. For example: *All XXX files are available from the XXX database (accession number(s) XXX, XXX.).*<br>• If the data are all contained **within the manuscript and/or Supporting Information files**, enter the following: *All relevant data are within the manuscript and its Supporting Information files.*<br>• If neither of these applies but you are able to provide **details of access elsewhere**, with or without limitations, please do so. For example:<br><br>*Data cannot be shared publicly because of [XXX]. Data are available from the XXX Institutional Data Access / Ethics*	All files are available from the GWAS database (accession number(s) ukb-b-19886, ieu-b-4905, ebi-a-GCST90006913, ebi-a-GCST90006914, ebi-a-GCST90006915, ebi-a-GCST90006916, ebi-a-GCST90006912, ebi-a-GCST90006911.).

Committee (contact via XXX) for researchers who meet the criteria for access to confidential data.<br><br>*The data underlying the results presented in the study are available from (include the name of the third party and contact information or URL).*<br>• This text is appropriate if the data are owned by a third party and authors do not have permission to share the data.<br><br>* typeset	
Additional data availability information:	

Jiaqin Chen & Junning Zhang-Manuscript Click here to access/download;Manuscript;Jiaqin Chen & Junning Zhang-Manuscript.docx

Causal Relationship Between Helicobacter pylori Antibodies and gastroesophageal reflux disease (GERD): A Mendelian Study

Jiaqin Chen, Affiliation 1: Graduate School, Beijing University of Chinese Medicine. Affiliation 2: Department of Gastroenterology, Dongzhimen Hospital, Beijing University of Chinese Medicine. Beijing, China, chikashion@163.com

Junning Zhang, Affiliation 1: Graduate School, Beijing University of Chinese Medicine. Affiliation 2: Department of Oncology of Integrative Chinese and Western Medicine, China-Japan Friendship Hospital, Beijing, China, junningzhang@foxmail.com

Jiaqin Chen# and Junning Zhang# contributed equally. Jiaqin Chen# and Junning Zhang# are **joint first authors**.

Xiaolu Ma, Affiliation 1: Graduate School, Beijing University of Chinese Medicine. Affiliation 2: Department of Gastroenterology, Dongzhimen Hospital, Beijing University of Chinese Medicine. Beijing, China, 765386073@qq.com

Yuehan Ren, Graduate School, Beijing University of Chinese Medicine, Beijing, China, 18810055853@163.com

Yi Tang, School of Traditional Chinese Medicine, Beijing University of Chinese Medicine, Beijing, China, bucmtangyi@163.com

Zhongmian Zhang, Affiliation 1: Graduate School, Beijing University of Chinese Medicine. Affiliation 2: Department of Gastroenterology, Dongzhimen Hospital, Beijing University of Chinese Medicine. Beijing, China, 1093225412@qq.com

Wangyu Ye, Affiliation 1: Graduate School, Beijing University of Chinese Medicine. Affiliation 2: Department of Gastroenterology, Dongzhimen Hospital, Beijing University of Chinese Medicine. Beijing, China, 1079043145@qq.com

Xiyan Zhang, Affiliation 1: Graduate School, Beijing University of Chinese Medicine. Affiliation 2: Department of Gastroenterology, Dongzhimen Hospital, Beijing University of Chinese Medicine. Beijing, China, 1843685817@qq.com

Zili Lin, Affiliation 1: Graduate School, Beijing University of Chinese Medicine. Affiliation 2: Department of Gastroenterology, Dongzhimen Hospital, Beijing University of Chinese Medicine. Beijing, China, 543646724@qq.com

Lan Wang, Affiliation 1: Graduate School, Beijing University of Chinese Medicine. Affiliation 2: Department of Gastroenterology, Dongzhimen Hospital, Beijing University of Chinese Medicine. Beijing, China, 626865909@qq.com

Zhihong Li*, MD., Ph.D., Department of Gastroenterology, Dongzhimen Hospital, Beijing University of Chinese Medicine. Beijing, China, lzhhsm@126.com

* Zhihong Li, MD., Ph.D., Professor

Affiliation: Department of Gastroenterology, Dongzhimen Hospital, Beijing University of Chinese Medicine. Beijing, China.

Correspondence address: Dongzhimen Hospital, Beijing University of Traditional Chinese Medicine, No. 5, Haiyuncang Hutong, Dongcheng District, Beijing, China

Email: lzhhsm@126.com

Phone number: +86 13521162969

Abstract

Background

Observational studies have indicated that both Helicobacter pylori infection and the presence of Helicobacter pylori antibodies may increase the risk of gastroesophageal reflux disease (GERD). However, the exact association between Helicobacter pylori antibodies and the occurrence of GERD remains largely unresolved. Therefore, this two-sample Mendelian randomization (MR) study aims to investigate the causal relationship between Helicobacter pylori infection and GERD.

Methods

This study encompassed seven different specific protein antibodies targeting Helicobacter pylori and utilized a genome-wide association study (GWAS) on GERD. MR analysis was conducted to assess the causal relationship between Helicobacter pylori antibodies and the development of GERD.

Results

Genetically predicted serum levels of Helicobacter pylori IgG antibodies were positively associated with an increased risk of GERD (odds ratio [OR] = 1.001, 95% CI 1.000-1.003, P = 0.043). No causal relationship was found between other Helicobacter pylori antibodies and gastroesophageal reflux disease.

Conclusion

The outcomes derived from our two-sample Mendelian randomization analysis demonstrate a discernible link between the levels of Helicobacter pylori IgG antibodies and an augmented susceptibility to GERD. However, it is imperative to expand the sample size further in order to corroborate the correlation between Helicobacter pylori infection and GERD.

Keywords

Mendelian randomization, Helicobacter pylori, gastroesophageal reflux disease, bidirectional, causal

Introduction

Gastroesophageal reflux disease (GERD) is a prevalent gastrointestinal disorder frequently encountered in clinical settings, particularly in Western countries. It affects approximately 20% of the population, making it a prevalent and widespread condition. GERD is known to cause distressing symptoms that significantly impact individuals' quality of life[1], GERD significantly compromises patients' quality of life and imposes a substantial economic burden[2], The clinical manifestations of GERD commonly include a burning sensation behind the sternum, characterized by the regurgitation of gastric contents into the throat, leading to mucosal damage of the affected organs[3], chronic GERD, if left untreated, can progress to Barrett's esophagus (BO), which represents a precancerous condition of the esophagus preceding the development of esophageal cancer[4].

Helicobacter pylori is a Gram-negative bacterium. It is widely believed that Helicobacter pylori infection impacts a substantial proportion of the global population, with a prevalence surpassing 60.3%. Economically disadvantaged regions experience even higher rates, exceeding 80% in some cases[5], Helicobacter pylori plays a vital role in gastric mucosal inflammation and the carcinogenesis process. The potential causal relationship between Helicobacter pylori infection and the occurrence of GERD is currently a topic of debate. Several studies suggest that Helicobacter pylori infection might be a contributing factor in the development of esophageal diseases. Further research is needed to fully understand the complex interaction between Helicobacter pylori and GERD[6]. A study involving 156 patients with peptic ulcers and reflux esophagitis found that eradication of Helicobacter pylori led to a significant improvement in gastroesophageal reflux symptoms compared to patients with persistent infection[7], however, there have been studies that arrived at the opposite conclusion, suggesting that Helicobacter pylori infection may actually serve as a protective factor for GERD[8]. It is important to note that all the evidence currently available is based on observational studies, which inherently have notable limitations and are susceptible to confounding factors, reverse causation, or other biases due to unmeasured or inaccurately measured variables. The causal association between Helicobacter pylori infection and GERD is still uncertain, as there is a dearth of conclusive evidence in this regard. This uncertainty is particularly significant in terms of the potential utilization of Helicobacter pylori eradication as a preventive or therapeutic approach for GERD. Further research is essential to gain a clearer understanding of the connection between Helicobacter pylori and GERD, enabling more informed decisions regarding treatment strategies.

Mendelian randomization (MR) is a robust method that utilizes genetic variations to assess whether the observed correlations between risk factors and outcomes are indicative of causal effects. This methodology capitalizes on the inherent randomness and natural assortment of genetic variations during meiosis, resulting in a randomized dispersion of genetic variants across the population[9], Genetic variation serves as an instrumental variable for the exposure, as it is randomly allocated during conception, thus minimizing residual confounding to the greatest extent possible[10]. The MR design offers a rigorous approach to elucidate causal relationships between complex diseases. The availability of genome-wide association study (GWAS) data has greatly enhanced the accessibility and widespread utilization of two-sample MR designs. In the current investigation, a two-sample MR analysis was employed to predict the causal link between Helicobacter pylori infection and GERD, thereby providing insightful implications for clinical utilization.

Study design

In the current investigation, a univariable Mendelian randomization analysis was utilized to explore

the causal influence of Helicobacter pylori infection on GERD and unveil plausible underlying mechanisms. The schematic representation of the study design can be found in Figure 1. The data used for this analysis were sourced from previously published GWAS. It is important to note that all original studies obtained ethical consent. Helicobacter pylori infection was defined based on the measurement of serum-specific antibodies targeting Helicobacter pylori proteins, incorporating data from seven different antibody measurements. The resultant dataset comprised 16,404 individuals from the IEU GWAS database, including levels of IgG, CagA, VacA, UREA, hydrogen peroxide enzyme, OMP, and GroEL antibodies against Helicobacter pylori. These data can be found on the IEU OpenGWAS project (mrcieu.ac.uk) and all study participants were of European ancestry. GERD candidate genes were extracted from the IEU GWAS database for this study, including 4,360 cases and 458,650 controls. MR analysis relies on three critical assumptions[11]. Assumption one posits that the instrumental variable, represented by genetic variation in this scenario, ought to exhibit a robust association with the focal exposure. Assumption two posits that the genetic variation should not be associated with any confounding factors that could potentially impact both the exposure and the outcome. Assumption three implies that the genetic variation affects the outcome exclusively through its direct influence on the exposure.

In this MR study, we employed single nucleotide polymorphisms (SNPs) obtained from GWAS as instrumental variables (IVs). Initially, we employed a p-value threshold of 5E-06 for IV selection, aiming to obtain a sufficient number of IVs and bolster statistical power. It is worth noting that this threshold has been commonly employed in comparable studies[12], furthermore, we conducted linkage disequilibrium (LD) clumping by aggregating genetic variants By applying a threshold of $R^2 < 0.001$ within a window size of 10,000 kb. To address potential horizontal pleiotropy, we manually selected proxies from the Pheno Scanner database (http://phenoscanner.medschl.cam.ac.uk) and assessed their association with any previously identified confounding traits ($p < 5E-08$). IVs associated with GERD were excluded. To address the issue of weak instrument bias and its influence on causal inference, we assessed the strength of instrumental variables (IVs) using the formula $F = b^2exposure / SE^2exposure$[13], SNPs with an F-statistic below 10 were deemed weak instrumental variables (IVs) and were excluded from the analysis[14]. We assessed the causal effects of GERD outcomes using five distinct approaches: weighted median, MR-Egger regression, inverse-variance weighted (IVW), weighted mode, and simple mode. The primary statistical model utilized in this investigation was the random-effects inverse-variance weighted (IVW) method, which effectively addresses heterogeneity[15], the IVW method combines the inverse of each IV's variance as weights, disregarding the intercept term in the regression, to yield a weighted average of the IV effects as the final outcome. In contrast, MR-Egger regression takes into account the inclusion of the intercept term in the regression and employs the inverse of the residual variance as weights for fitting. The weighted median estimator (WME) represents the median value derived from the weighted empirical density function of the ratio estimate in question. which represents the consistently estimated causal relationship by half of the valid instrumental variables utilized in the analysis.

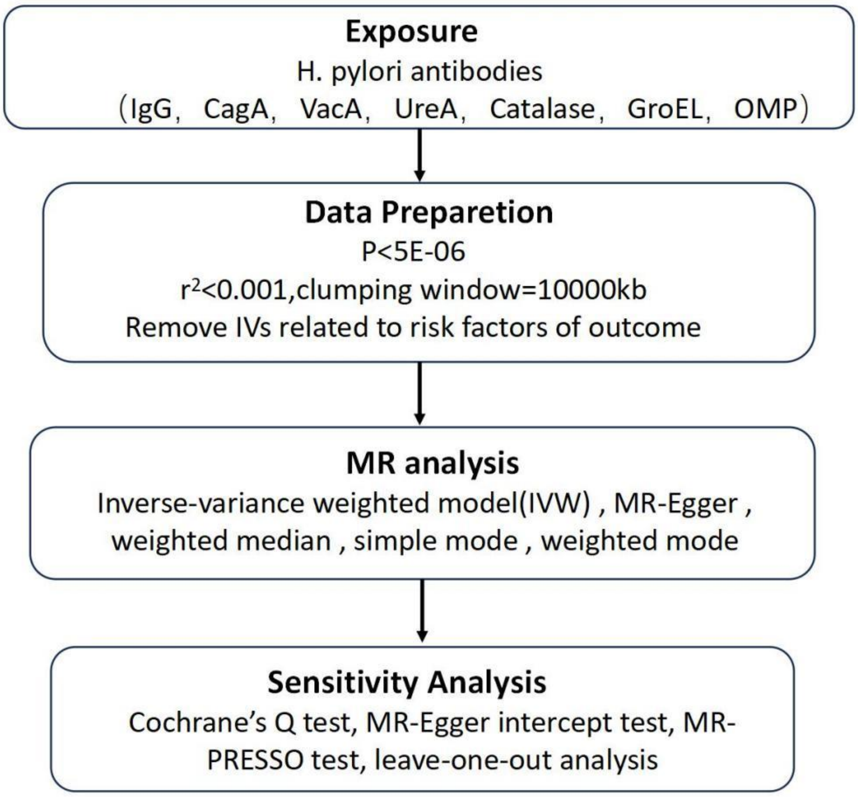

Figure 1: Flow chart

Sensitivity Analysis

Due to potential heterogeneity arising from different data sources, MR analysis may introduce bias in the estimation of causal effects. An extensive sensitivity analysis was conducted to assess the robustness and reliability of the MR results, ensuring the validity of the findings in the face of potential confounding factors. Within this study, the primary instrumental variable weighted (IVW) and Mendelian randomization Egger (MR-Egger) methods were evaluated for heterogeneity. The presence of heterogeneity was evaluated using Cochran's Q test, a widely utilized statistical method in medical research. This test allowed for the assessment and quantification of potential variations and discrepancies among the analyzed data, contributing to a comprehensive understanding of the study's findings. To assess the credibility of the causal estimates, the sensitivity analysis encompassed a range of methodologies in the present study. These included the utilization of the MR-Egger intercept test, and leave-one-out analysis. By incorporating these rigorous approaches, the research aimed to comprehensively evaluate and validate the robustness of the causal inferences derived from the MR framework. This comprehensive sensitivity analysis bolstered the reliability and confidence in the study's findings, enhancing its contribution to the field of medicine. Statistical analyses and data visualizations were performed utilizing R version 4.2.1. Mendelian randomization analyses were performed with the use of the Two Sample MR Package, version 0.5.6.

Result

To investigate the causal association between Helicobacter pylori and the risk of GERD, this study employed a comprehensive utilization of GWAS meta-analysis dat[illegible]he incorporation of this extensive dataset allowed for a thorough exploration of the genetic factors underlying both

Helicobacter pylori infection and GERD susceptibility. It is contributing to our understanding of the pathophysiology and etiology of this prevalent gastrointestinal disorder It represents the first study exploring the potential causal relationship between Helicobacter pylori and GERD. Our MR analysis demonstrated a causal link between genetically predicted levels of Helicobacter pylori IgG antibodies and the occurrence of GERD, although the findings were not entirely consistent. Using five different methods to analyze the antibody levels of Helicobacter pylori IgG, CagA, VacA, UREA, hydrogen peroxide enzyme, OMP, and GroEL, under the IVW method, an increased risk of GERD was associated with Helicobacter pylori IgG antibody levels (odds ratio [OR] = 1.003, 95% CI 1.00–1.004, P = 0.043) (See Figure 2). In order to address the potential influence of pleiotropy, one important tests were performed: the MR-Egger intercept test. The MR-Egger analysis, a statistical method designed to detect potential horizontal pleiotropy, was conducted to examine whether there were any genetic variants influencing both the exposure (Helicobacter pylori infection) and the outcome (GERD) through pathways unrelated to the causal relationship of interest. Remarkably, the results of the MR-Egger intercept test exhibited no significant evidence of horizontal pleiotropy, as indicated by all P-values surpassing the threshold of 0.05. This outcome suggests that the instrumental variables employed in the MR analysis were not biased by horizontal pleiotropy, reinforcing the credibility of the causal inference made regarding the association between Helicobacter pylori infection and GERD risk. Cochran's Q test and I^2 statistics indicated low heterogeneity and high reliability of these SNPs (all $P > 0.05$). The funnel plot exhibited general symmetry, suggesting limited evidence of heterogeneity. (Refer to the Supplement materials)

Exposure	Method	N SNP	MR analysis		Heterogeneity test		MR-Egger regression	
			OR (95%CI)	P value	Cochran's Q	P value	Egger intercept	P value
HP-IgG	MR Egger	7	1.007(0.990-1.014)	0.147	1.819390	0.610	-0.0008	0.224
	Weighted median	7	1.002 (0.999-1.004)	0.074				
	IVW	7	1.001(1.000-1.003)	0.043	4.146256	0.386		
	Simple mode	7	1.002(0.999-1.005)	0.176				
	Weighted mode	7	1.002(0.999-1.005)	0.174				
CagA	MR Egger	3	0.996(0.983-1.009)	0.713	0.1791725	0.672	0.0009	0.651
	Weighted median	3	1.000(0.999-1.002)	0.427				
	IVW	3	1.000(0.999-1.001)	0.161	0.5510087	0.759		
	Simple mode	3	1.000(0.998-1.002)	0.595				
	Weighted mode	3	1.000(0.998-1.002)	0.623				
VacA	MR Egger	5	0.997(0.987- 1.007)	0.63	9.856508	0.019	0.0006	0.589
	Weighted median	5	1.000(0.999- 1.002)	0.411				
	IVW	5	1.000(0.998- 1.002)	0.732	11.048687	0.026		
	Simple mode	5	1.000(0.998- 1.002)	0.556				
	Weighted mode	5	1.000(0.998- 1.002)	0.507				
Urea	MR Egger	4	0.996(0.987-1.005)	0.516	1.143195	0.564	0.0006	0.496
	Weighted median	4	1.000(0.998-1.001)	0.843				
	IVW	4	1.000(0.998-1.001	0.861	1.810789	0.612		
	Simple mode	4	1.000(0.998-1.002)	0.779				
	Weighted mode	4	1.000(0.997-1.002)	0.856				
Catalase	MR Egger	3	0.987(0.961-1.013)	0.523	0.2561435	0.612	0.002	0.536
	Weighted median	3	0.999(0.998-1.001)	0.888				
	IVW	3	0.999(0.998-1.000)	0.467	1.0509169	0.591		
	Simple mode	3	0.999(0.997-1.002)	0.987				
	Weighted mode	3	1.000(0.997-1.002)	0.999				
OMP	MR Egger	3	0.997(0.987-1.006)	0.655	0.8282778	0.363	0.0004	0.744
	Weighted median	3	0.999(0.997-1.001)	0.518				
	IVW	3	0.999(0.997-1.000)	0.288	1.0087270	0.604		
	Simple mode	3	0.999(0.997-1.002)	0.936				
	Weighted mode	3	0.999(0.997-1.002)	0.938				
GroEL	MR Egger	3	0.957(0.957-1.046)	0.963	5.168425	0.023	0.0030	0.951
	Weighted median	3	0.998(0.998-1.003)	0.499				
	IVW	3	0.996(0.996-1.002)	0.773	5.199087	0.074		
	Simple mode	3	0.997(0.997-1.004)	0.547				
	Weighted mode	3	0.997(0.997-1.004)	0.549				

Table 1: Causal relationship between Helicobacter pylori antibodies and GERD

Exposure	Methods	no.SNPs	P-value	OR(95%CI)
HP-IgG	MR Egger	7	0.147	1.007(0.990,1.014)
	Weighted median	7	0.074	1.002(0.999,1.004)
	Inverse variance weighted	7	0.043	1.001(1.000,1.003)
	Simple mode	7	0.176	1.002(0.999,1.005)
	Weighted mode	7	0.174	1.002(0.999,1.005)
CagA	MR Egger	3	0.713	0.996(0.983,1.009)
	Weighted median	3	0.427	1.000(0.999,1.002)
	Inverse variance weighted	3	0.161	1.000(0.999,1.001)
	Simple mode	3	0.595	1.000(0.998,1.002)
	Weighted mode	3	0.623	1.000(0.998,1.002)
VacA	MR Egger	5	0.63	0.997(0.987, 1.007)
	Weighted median	5	0.411	1.000(0.999, 1.002)
	Inverse variance weighted	5	0.732	1.000(0.998, 1.002)
	Simple mode	5	0.556	1.000(0.998, 1.002)
	Weighted mode	5	0.507	1.000(0.998, 1.002)
Urea	MR Egger	4	0.516	0.996(0.987,1.005)
	Weighted median	4	0.843	1.000(0.998,1.001)
	Inverse variance weighted	4	0.861	1.000(0.998,1.001
	Simple mode	4	0.779	1.000(0.998,1.002)
	Weighted mode	4	0.856	1.000(0.997,1.002)
Catalase	MR Egger	3	0.523	0.987(0.961,1.013)
	Weighted median	3	0.888	0.999(0.998,1.001)
	Inverse variance weighted	3	0.467	0.999(0.998,1.000)
	Simple mode	3	0.987	0.999(0.997,1.002)
	Weighted mode	3	0.999	1.000(0.997,1.002)
OMP	MR Egger	3	0.655	0.997(0.987,1.006)
	Weighted median	3	0.518	0.999(0.997,1.001)
	Inverse variance weighted	3	0.288	0.999(0.997,1.000)
	Simple mode	3	0.936	0.999(0.997,1.002)
	Weighted mode	3	0.938	0.999(0.997,1.002)
GroEL	MR Egger	3	0.963	0.957(0.957,1.046)
	Weighted median	3	0.499	0.998(0.998,1.003)
	Inverse variance weighted	3	0.773	0.996(0.996,1.002)
	Simple mode	3	0.547	0.997(0.997,1.004)
	Weighted mode	3	0.549	0.997(0.997,1.004)

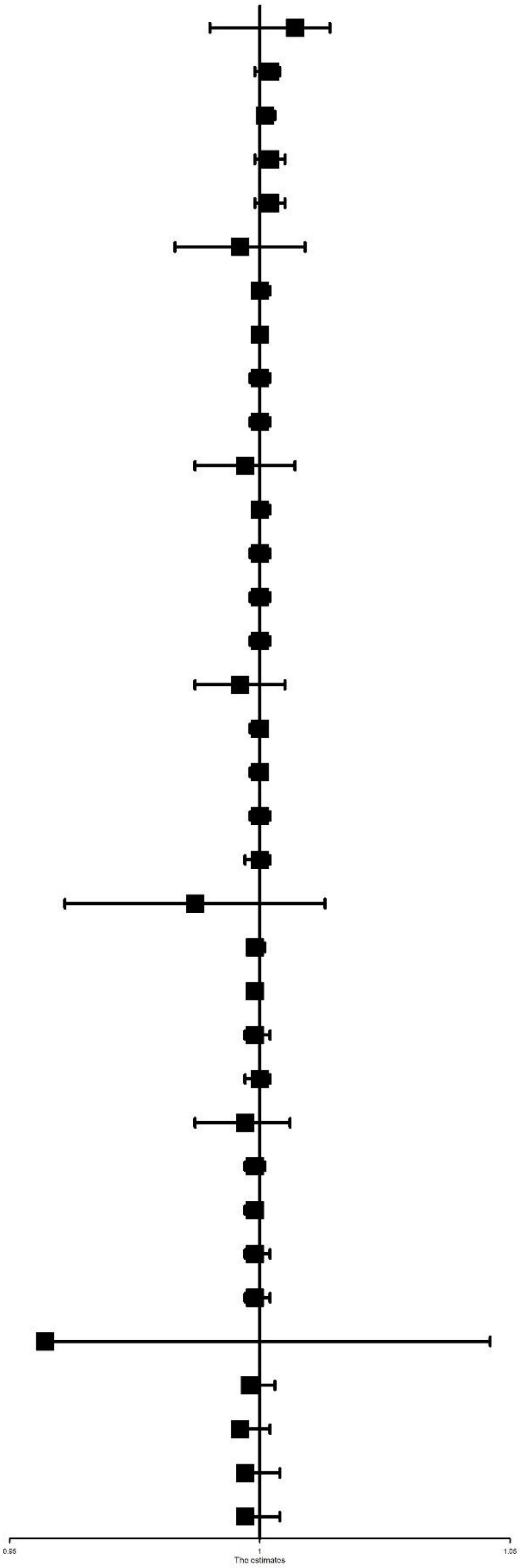

Figure 2: Forest plot

Discussion

The association between Helicobacter pylori infection and GERD continues to be a subject of

ongoing debate and scientific inquiry, and currently, there is no definitive causal relationship established. Although previous observational studies have been limited, the complexity of this relationship arises from various factors, including the heterogeneity of study populations, differences in study designs, and the multifactorial nature of GERD etiology, several studies[16–20] have found an increased risk of GERD in both Eastern and Western populations with Helicobacter pylori infection, they have reported an elevated incidence of GERD associated with Helicobacter pylori infection, the current MR study supports this finding. Emerging evidence from multiple studies has consistently demonstrated a robust correlation between Helicobacter pylori infection and the heightened risk of GERD[21], furthermore, scientific research has established a correlation between Helicobacter pylori infection and mild GERD. These findings underscore the association between Helicobacter pylori infection and the presence of mild GERD symptoms[22], in a randomized controlled study, it was observed that individuals with Helicobacter pylori infection exhibited a higher prevalence of heartburn symptoms in comparison to those without Helicobacter pylori infection[23], The findings of this study indicate an elevated likelihood of experiencing symptoms of heartburn in association with Helicobacter pylori infection. The presence of Helicobacter pylori infection appears to be associated with an increased susceptibility to developing or experiencing symptoms of heartburn. The malfunctioning of the lower esophageal sphincter is considered the primary contributor to the development of GERD. Simultaneously, the gastric mucosa produces a variety of cytokines which activate immune cell recruitment and migration and contribute to the development of the disease[24]. Multiple theoretical mechanisms are implicated in the development of GERD caused by Helicobacter pylori infection. Certain studies propose that Helicobacter pylori infection can impact the secretion of gastric acid[23], Considering that Helicobacter pylori infection primarily elicits gastritis, it leads to increased gastric acid secretion, resulting in increased acid exposure to the lower esophagus, leading to symptoms such as acid reflux and heartburn. Additional research has revealed that the existence of Helicobacter pylori hampers the mucosal healing capacity among individuals with reflux esophagitis, thereby intensifying symptoms of reflux[26], Moreover, prospective studies have unequivocally revealed a compelling association between Helicobacter pylori infection and a notable escalation in inflammatory processes transpiring at the delicate juncture between the esophagus and the stomach among patients afflicted with gastroesophageal reflux[27].

This study has several limitations. Firstly, the sample size utilized in the GWAS pertaining to Helicobacter pylori infection was relatively modest in scale, which may introduce some bias and warrants further investigation with larger sample sizes. Secondly, a p-value threshold of 5E-06 is employed for instrumental variable selection, with the potential to introduce a certain degree of weak instrument bias that can impact the overall estimation. Thirdly, the scope of our study was confined to individuals with European ancestry, thereby potentially imposing constraints on the applicability of our findings to broader populations.

Conclusion

Based on the comprehensive analysis of our study data, compelling evidence emerges to support a robust causal nexus between the genetically predicted levels of Helicobacter pylori IgG antibodies and a discernible augmentation in the vulnerability to GERD. However, it is imperative to underscore that additional endeavors encompassing more expansive cohorts are imperative to corroborate and fortify the intricate interplay between Helicobacter pylori infection and the

manifestation of GERD. The pursuit of future research endeavors, characterized by enlarged sample sizes, shall undoubtedly engender a deeper understanding of the intricate pathophysiological mechanisms underpinning this intriguing association.

Funding

Transverse project of Dongzhimen Hospital of Beijing University of Traditional Chinese Medicine: Improvement effect of Lactobacillus plantarum HCS-001 on gastric discomfort symptoms in Helicobacter pylori infected patients (No. HX-DZM-202269).

Supporting information

Acknowledgments

We would like to thank all participants and investigators who contributed to the GWAS data.

Author Contributions

Formal analysis: Junning Zhang**.**

Writing – original draft: Jiaqin Chen**.**

Writing – review & editing: Xiaolu Ma, Yuehan Ren, Yi Tang, Zhongmian Zhang, Wangyu Ye, Xiyan Zhang, Zili Lin, Lan Wang, Zhihong Li.

Consent for publication

All authors consent and approve the manuscript for publication.

Competing interests

None declared.

References

1. Peery, A. F. *et al.* Burden and Cost of Gastrointestinal, Liver, and Pancreatic Diseases in the United States: Update 2018. *Gastroenterology* **156**, 254-272.e11 (2019).
2. Eusebi, L. H. *et al.* Global prevalence of, and risk factors for, gastro-oesophageal reflux symptoms: a meta-analysis. *Gut* **67**, 430–440 (2018).
3. Maret-Ouda, J., Markar, S. R. & Lagergren, J. Gastroesophageal Reflux Disease. *JAMA* **324**, 2565 (2020).
4. Spechler, S. J. Barrett esophagus and risk of esophageal cancer: a clinical review. *JAMA* **310**, 627–636 (2013).

5. Malfertheiner, P. *et al.* Management of Helicobacter pylori infection-the Maastricht V/Florence Consensus Report. *Gut* **66**, 6–30 (2017).

6. Rubenstein, J. H. *et al.* Association between Helicobacter pylori and Barrett's esophagus, erosive esophagitis, and gastroesophageal reflux symptoms. *Clin. Gastroenterol. Hepatol. Off. Clin. Pract. J. Am. Gastroenterol. Assoc.* **12**, 239–245 (2014).

7. Ishiki, K. *et al.* Helicobacter pylori eradication improves pre-existing reflux esophagitis in patients with duodenal ulcer disease. *Clin. Gastroenterol. Hepatol. Off. Clin. Pract. J. Am. Gastroenterol. Assoc.* **2**, 474–479 (2004).

8. Zhao, T., Liu, F. & Li, Y. Effects of Helicobacter pylori eradication on esophageal motility, esophageal acid exposure, and gastroesophageal reflux disease symptoms. *Front. Cell. Infect. Microbiol.* **13**, 1082620 (2023).

9. Emdin, C. A., Khera, A. V. & Kathiresan, S. Mendelian Randomization. *JAMA* **318**, 1925–1926 (2017).

10. Bowden, J. & Holmes, M. V. Meta-analysis and Mendelian randomization: A review. *Res. Synth. Methods* **10**, 486–496 (2019).

11. Burgess, S., Small, D. S. & Thompson, S. G. A review of instrumental variable estimators for Mendelian randomization. *Stat. Methods Med. Res.* **26**, 2333–2355 (2017).

12. Guo, X., Tang, P., Hou, C., Liu, Y. & Li, R. Impaired pulmonary function mediates the impact of preterm birth on later-life stroke: a 2-step, multivariable Mendelian randomization study. *Epidemiol. Health* **45**, e2023031 (2023).

13. Zhang, Y. *et al.* Causal associations between gut microbiome and cardiovascular disease: A Mendelian randomization study. *Front. Cardiovasc. Med.* **9**, 971376 (2022).

14. Bowden, J. *et al.* Assessing the suitability of summary data for two-sample Mendelian randomization analyses using MR-Egger regression: the role of the I2 statistic. *Int. J. Epidemiol.* **45**, 1961–1974 (2016).

15. Hemani, G. *et al.* The MR-Base platform supports systematic causal inference across the human phenome. *eLife* **7**, e34408 (2018).

16. Usui, G. *et al.* Association Between Helicobacter pylori Infection and Short-segment/Long-segment Barrett's Esophagus in a Japanese Population: A Large Cross-Sectional Study. *J. Clin. Gastroenterol.* **54**, 439–444 (2020).

17. Jonaitis, L. V., Kiudelis, G. & Kupcinskas, L. Characteristics of patients with erosive and nonerosive GERD in high-Helicobacter-pylori prevalence region. *Dis. Esophagus Off. J. Int. Soc. Dis. Esophagus* **17**, 223–227 (2004).

18. Wu, J. C. Y., Cheung, C. M. Y., Wong, V. W. S. & Sung, J. J. Y. Distinct clinical characteristics between patients with nonerosive reflux disease and those with reflux esophagitis. *Clin. Gastroenterol. Hepatol. Off. Clin. Pract. J. Am. Gastroenterol. Assoc.* **5**, 690–695 (2007).

19. Ronkainen, J. *et al.* High prevalence of gastroesophageal reflux symptoms and esophagitis with or without symptoms in the general adult Swedish population: a Kalixanda study report. *Scand. J. Gastroenterol.* **40**, 275–285 (2005).

20. Dore, M. P. *et al.* Risk factors for erosive and non-erosive gastroesophageal reflux disease and Barrett's esophagus in Nothern Sardinia. *Scand. J. Gastroenterol.* **51**, 1281–1287 (2016).

21. Sharifi, A., Dowlatshahi, S., Moradi Tabriz, H., Salamat, F. & Sanaei, O. The Prevalence, Risk Factors, and Clinical Correlates of Erosive Esophagitis and Barrett's Esophagus in Iranian Patients with Reflux Symptoms. *Gastroenterol. Res. Pract.* **2014**, 696294 (2014).

22. Wu, J. C. *et al.* Helicobacter pylori infection is associated with milder gastro-oesophageal reflux disease. *Aliment. Pharmacol. Ther.* **14**, 427–432 (2000).

23. Harvey, R. F. *et al.* Randomised controlled trial of effects of Helicobacter pylori infection and its eradication on heartburn and gastro-oesophageal reflux: Bristol helicobacter project. *BMJ* **328**, 1417 (2004).

24. Altomare, A., Guarino, M. P. L., Cocca, S., Emerenziani, S. & Cicala, M. Gastroesophageal reflux disease: Update on inflammation and symptom perception. *World J. Gastroenterol. WJG* **19**, 6523–6528 (2013).

25. el-Omar, E. M. *et al.* Helicobacter pylori infection and abnormalities of acid secretion in patients with duodenal ulcer disease. *Gastroenterology* **109**, 681–691 (1995).

26. Malfertheiner, P. *et al.* Prognostic influence of Barrett's oesophagus and Helicobacter pylori infection on healing of erosive gastro-oesophageal reflux disease (GORD) and symptom resolution in non-erosive GORD: report from the ProGORD study. *Gut* **54**, 746–751 (2005).

27. Pieramico, O. & Zanetti, M. V. Relationship between intestinal metaplasia of the gastro-oesophageal junction, Helicobacter pylori infection and gastro-oesophageal reflux disease: a prospective study. *Dig. Liver Dis. Off. J. Ital. Soc. Gastroenterol. Ital. Assoc. Study Liver* **32**, 567–572 (2000).

Figure 1

Click here to access/download;Figure;Figure 1.png

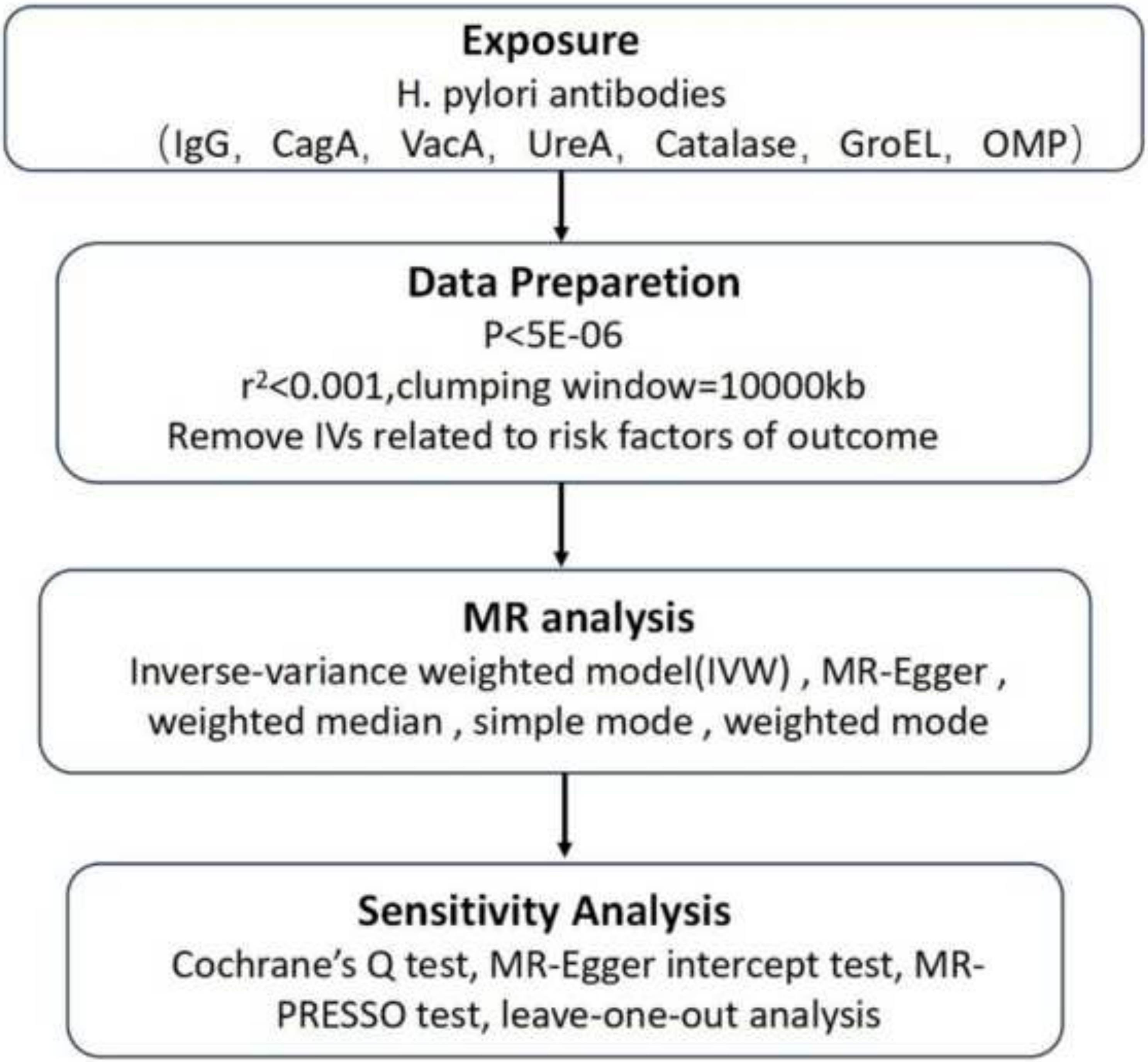

Figure 2

Click here to access/download;Figure;Figuer 2.png

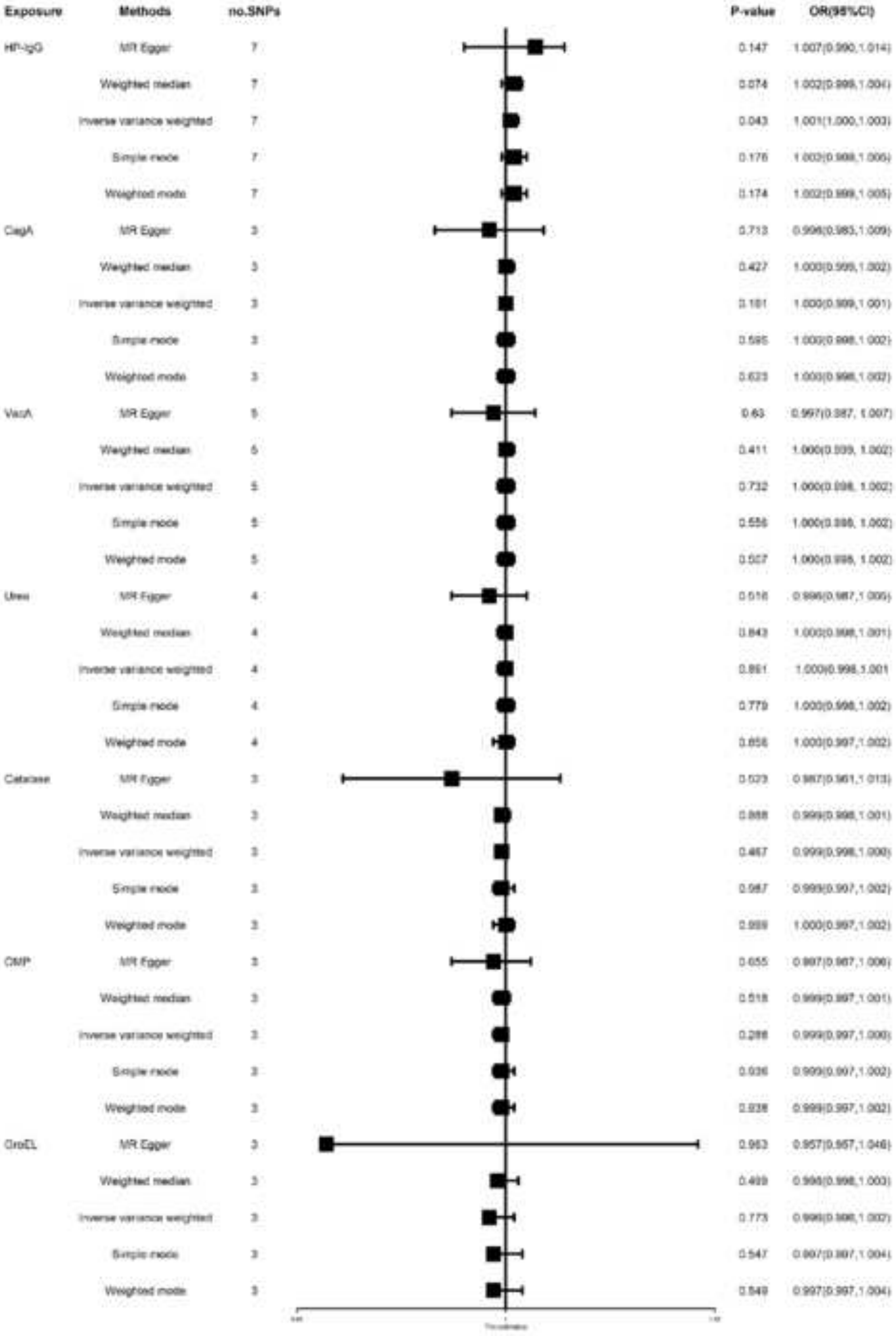

Click here to access/download
Supporting Information
Supporting Information.docx
