# Supplementary material for: Causal relationship between Helicobacter pylori antibodies and gastroesophageal reflux disease (GERD): A mendelian study
Source: PLoS One. 2023 Dec 11;18(12):e0294771. doi: 10.1371/journal.pone.0294771 (PMC10712878; doi:10.1371/journal.pone.0294771)
Supplement: Attachment — Submitted filename: Response to Reviewers.docx [file pone.0294771.s007.docx]

Dear Editor and Reviewers,

Thank you for giving us the opportunity to revise our manuscript "Causal Relationship Between Helicobacter pylori Antibodies and gastroesophageal reflux disease (GERD): A Mendelian Study" (Submission ID PONE-D-23-28111). We gratefully appreciate the valuable comments and suggestions provided by the reviewers, and we believe that their input has greatly improved our manuscript.

We have carefully considered the reviewers’ suggestions and have made comments and revisions to address all their concerns. Our responses to the editor’s and reviewers’ comments are noted below (marked with ‘>>Reply:’ and highlighted in blue). In the revised manuscript, red highlighted text indicates where changes have been made. We have responded to all the issues identified in document “PONE-D-23-28111_reviewer-REVISION”, as detailed in “PONE-D-23-28111_author-REVISION”.

We hope this revised version is acceptable for publication.

On behalf of all authors,

Jiaqin Chen & Junning Zhang

Corresponding Author:

Zhihong Li*

Email Address: lzhhsm@126.com

MD., Ph.D.; Professor

Department of Gastroenterology,

Dongzhimen Hospital, Beijing University of Chinese Medicine. Beijing, China.

Journal Requirements and Editor Comments:

Thanks to the authors for the study.

I invite you to resubmit your manuscript after addressing two reviewers’ comments. When resubmitting your manuscript, please carefully consider all issues mentioned in the reviewers' comments, outline every change made point by point, and provide suitable rebuttals for any comments not addressed.

**>>Reply:**

Thank you for giving us the opportunity to revise our manuscript. We have carefully considered your and reviewers’ suggestions. In our revision, we have resubmitted our manuscript as required by the template provided on the official website. The funder's role in the study, Competing Interests and Supporting Information have been added to our cover letter.

In addition, we have carefully response all the other comments from reviewers.

Reviewer comments

**Introduction**

ıntroduction section was written so long. It must be shortened.

**>>Reply:**

Thank you for your suggestion. We have cut the introduction part as required, see 'Revised Manuscript with Track Changes' for details.

Authors should clearly explain the aims of the study.

**>>Reply:**

Thank you for your comments. We have added in our manuscript. Details are as follows, "*the aim of this study is to investigate whether current or past Helicobacter pylori infection increases the risk of developing GERD, and to assess the potential of Helicobacter pylori i eradication as a preventive or therapeutic approach for GERD. The findings of this study will provide valuable recommendations for clinical applications and treatment strategies.*"

**Study Design**

What is type of the study ? prospective ? retospective ? cross-sectional ? ıt should be addreesed in beginning paragraphy.

**>>Reply:**

Mendelian Randomization (MR) is a data analysis technique for assessing etiological inferences in epidemiological studies that uses genetic variants with strong correlations with exposure factors as instrumental variables to assess causal relationships between exposure factors and outcomes. We have added the relevant content at beginning paragraphy as detailed below: “*MR Analysis is a type of instrumental variable analysis that uses genetic variants as instrumental variables. This methodology capitalizes on the inherent randomness and natural assortment of genetic variations during meiosis, resulting in a randomized dispersion of genetic variants across the population [1], Genetic variation serves as an instrumental variable for the exposure, as it is randomly allocated during conception, thus minimizing residual confounding to the greatest extent possible [2]. The MR design offers a rigorous approach to elucidate causal relationships between complex diseases. The availability of genome-wide association study (GWAS) data has greatly enhanced the accessibility and widespread utilization of two-sample MR designs.*”

Reference:

1.	Emdin, C. A., Khera, A. V. & Kathiresan, S. Mendelian Randomization. JAMA 318, 1925–1926 (2017).

2.	Bowden, J. & Holmes, M. V. Meta-analysis and Mendelian randomization: A review. Res. Synth. Methods 10, 486–496 (2019).

I wonder where authors obtained ethic approvement

**>>Reply:**

Thank you for your valuable comments. This study used publicly available de-identified data from participant studies that were approved by an ethical standards committee concerning human experimentation. No separate ethical approval was required in this study.

We have added this statement to our “Ethics statement”.

authors should mention where the study was conducted

**>>Reply:**

Thank you for your advice. We have already mentioned the software used in this study in "Sensitivity Analysis". See below for details: "*Statistical analyses and data visualizations were performed utilizing R version 4.2.1. Mendelian randomization analyses were performed with the use of the Two Sample MR Package, version 0.5.6.*"

some knowledge is required like how gerd was defined ? what methods were used on diagnosing GERD ?

**>>Reply:**

Thank you for your suggestions.

Q1: We have already covered GERD in detail in our introduction, see below: "*Gastroesophageal reflux disease (GERD) is a prevalent gastrointestinal disorder frequently encountered in clinical settings, particularly in Western countries. It affects approximately 20% of the population and nearly 40% of children [1], making it a prevalent and widespread condition. GERD is known to cause distressing symptoms that significantly impact individuals' quality of life [2], GERD significantly compromises patients' quality of life and imposes a substantial economic burden [3], The clinical manifestations of GERD commonly include a burning sensation behind the sternum, characterized by the regurgitation of gastric contents into the throat, leading to mucosal damage of the affected organs [4], three phenotypic presentations of GERD exist. Including non-erosive reflux disease (NERD), erosive oesophagitis (EE) and BE [5]. BE represents a precancerous condition of the esophagus preceding the development of esophageal cancer [6].*"

Reference:

1. Sahin, Y., Gubur, O. & Tekingunduz, E. Relationship between the severity of Helicobacter pylori infection and neutrophil and lymphocyte ratio and mean platelet volume in children. Arch. Argent. Pediatr. 118, e241–e245 (2020).

2. Peery, A. F. et al. Burden and Cost of Gastrointestinal, Liver, and Pancreatic Diseases in the United States: Update 2018. Gastroenterology 156, 254-272.e11 (2019).

3. Eusebi, L. H. et al. Global prevalence of, and risk factors for, gastro-oesophageal reflux symptoms: a meta-analysis. Gut 67, 430–440 (2018).

4. Maret-Ouda, J., Markar, S. R. & Lagergren, J. Gastroesophageal Reflux Disease. JAMA 324, 2565 (2020).

5. Fass, R. et al. Gastro-oesophageal reflux disease. Nat. Rev. Dis. Primer 7, 55 (2021).

6. Spechler, S. J. Barrett esophagus and risk of esophageal cancer: a clinical review. JAMA 310, 627–636 (2013).

Q2: Since you raised the issue of introduction being too long, we did not put this diagnosis in the manuscript, and explain it as follows: "GERD is empirically diagnosed and treated in clinical practice based on the clinician’s symptom assessment. Indications for testing include treatment failure, diagnostic uncertainty and treating (or preventing) complications of GERD. However, diagnostic testing may or may not support the initial diagnosis, as the criteria defining GERD are specific to each testing modality. GERD diagnostic methods include: clinical history and questionnaires, proton pump inhibitor trial, endoscopy and biopsy, ambulatory reflux monitoring, reflux-symptom association, oesophageal high-resolution manometry [1]."

Reference:

1. Gyawali CP, Kahrilas PJ, Savarino E, Zerbib F, Mion F, Smout AJPM, Vaezi M, Sifrim D, Fox MR, Vela MF, Tutuian R, Tack J, Bredenoord AJ, Pandolfino J, Roman S. Modern diagnosis of GERD: the Lyon Consensus. Gut. 2018 Jul;67(7):1351-1362.

ıt ıs required monre knowledge on participants

**>>Reply:**

Thank you for your comments, the open databases we use contain the following demographic information: race and gender, which has been supplemented with gender information in the "Study design".

**Sensitivity Analysis**

I could not see control group. If not present, authors should explaine why control group is absent .

**>>Reply:**

Thank you for your advice. The control group information in the database we used includes ethnicity and gender, as detailed in "*all study participants were of European ancestry, both men and women were included.*" in Study Design.

were the datas you founs showed normal distribution ?

**>>Reply:**

Thank you for your valuable comments. The methodology used in this study is suitable for genetic database studies and cannot be tested for normal distribution. The method of data analysis used in this study is independent of whether the data are normally distributed or not. Mendelian randomization (MR) is a method of causal inference based on genetic variation, the basic principle of which is to utilize the effect of randomly assigned genotypes on phenotypes in nature to infer the effect of biological factors on disease.

**Result**

I know what you investigate in the study. I think it is not needed writing it again and again

**>>Reply:**

Thank you for your suggestion. We have removed it from the manuscript.

the sentences is really redundant. the knowledge was written many times above

**>>Reply:**

I appreciate your recommendation. It is no longer included in the manuscript.

**Discussion**

the sentences from beginning discussion to 23 references give us same information that the association between Helicobacter pylori infection and GERD.

Author should enter by writing what they found their study..

**>>Reply:**

Thank you for your suggestion. We have described the findings of the references in more detail as follows: "in a Japanese population, Helicobacter pylori infection was significantly associated with short-segment Barrett's esophagus (SSBE), it may be a risk factor for SSBE [1]. At the same time, three studies conducted in Lithuania [2], Hong Kong [3], and the United States [4] respectively demonstrated that Helicobacter pylori is a statistically significant predictive factor for NERD. A study [5] conducted in Sweden revealed that adults infected with Helicobacter pylori (HP) in two cities in Sweden have a higher risk of developing symptoms of gastroesophageal reflux disease. An Iranian study showed that Helicobacter pylori infection is a significant and correlated risk factor for esophagitis [6]."

Reference:

1. Usui, G. et al. Association Between Helicobacter pylori Infection and Short-segment/Long-segment Barrett’s Esophagus in a Japanese Population: A Large Cross-Sectional Study. J. Clin. Gastroenterol. 54, 439–444 (2020).

2. Jonaitis, L. V., Kiudelis, G. & Kupcinskas, L. Characteristics of patients with erosive and nonerosive GERD in high-Helicobacter-pylori prevalence region. Dis. Esophagus Off. J. Int. Soc. Dis. Esophagus 17, 223–227 (2004).

3. Wu, J. C. Y., Cheung, C. M. Y., Wong, V. W. S. & Sung, J. J. Y. Distinct clinical characteristics between patients with nonerosive reflux disease and those with reflux esophagitis. Clin. Gastroenterol. Hepatol. Off. Clin. Pract. J. Am. Gastroenterol. Assoc. 5, 690–695 (2007).

4. Dore, M. P. et al. Risk factors for erosive and non-erosive gastroesophageal reflux disease and Barrett’s esophagus in Nothern Sardinia. Scand. J. Gastroenterol. 51, 1281–1287 (2016).

5. Ronkainen, J. et al. High prevalence of gastroesophageal reflux symptoms and esophagitis with or without symptoms in the general adult Swedish population: a Kalixanda study report. Scand. J. Gastroenterol. 40, 275–285 (2005).

6. Sharifi, A., Dowlatshahi, S., Moradi Tabriz, H., Salamat, F. & Sanaei, O. The Prevalence, Risk Factors, and Clinical Correlates of Erosive Esophagitis and Barrett’s Esophagus in Iranian Patients with Reflux Symptoms. Gastroenterol. Res. Pract. 2014, 696294 (2014).

authors should compare their results with other or similar studied. It was not adequately discussed their results. I think this may be limitation.

**>>Reply:**

Thank you for your comments. We have explained this in more detail in the manuscript, see below: "*Our MR study supports a direct relationship between Helicobacter pylori infection and GERD, MR Study enable the derivation of more robust and evidence-based conclusions, offering advantages over other methods without these issues. Our study is the first to reveal a causal relationship between H. pylori infection and GERD, and this study can improve the understanding of the pathogenic factors of GERD from a systems biology perspective.*"

**Conclusion**

Helicobacter pylori IgG antibody is so common among both adult and children. According your results, could it be thought predicted levels of helicobacter pylori IgG is linked GERD?

**>>Reply:**

Thank you for your suggestion. Helicobacter pylori IgG antibody is so common among both adult and children. From the results of Mendelian randomization, there is a causal relationship, but there is still a need for higher quality clinical studies or RCT trials to confirm this conclusion.

Reviewer's comments in the email
Reviewer #1

- All of the manuscript should be checked n terms of spelling errors.

**>>Reply:**

Thanks for your suggestion. We have carefully checked in current version.

- The manuscript appropriately cites the important and authoritative references but does not cite the recent published articles about Helicobacter pylori. If the recent published article about celiac disease for example ‘’Relationship between the severity of Helicobacter pylori infection and neutrophil and lymphocyte ratio and mean platelet volume in children. Arch Argent Pediatr 2020 Jun; 118(3): e241-e245’’ are cited, the manuscript would be better. The authors can benefit from this recent study.

**>>Reply:**

Thank you for your suggestions and for helping us with our research, we have cited this literature at introduction in the article.

Reviewer #2

Some revisions are required. ıntroduction was written so long. ıt should be shortened. I could not find ethical approval number and its year. some statistical informations should be added. In addition the finding of the study was not compared adequately with similar studies.

**>>Reply:**

Thank you for your advice. We have shortened the introduction, see the manuscript for details. Ethical and statistical information has been modified in the article. Specific information on the databases used in our study is specified in section of the article, for details see “Supporting Information”, our study is the first to reveal a causal relationship between H. pylori infection and GERD, and this study can improve the understanding of the pathogenic factors of GERD from a systems biology perspective.
